# Effects of Hypertonic Saline and Hydroxyethyl Starch on Myeloid-Derived Suppressor Cells in Hemorrhagic Shock Mice under Secondary Bacterial Attack

**DOI:** 10.1155/2020/5417201

**Published:** 2020-03-09

**Authors:** Jiu-Kun Jiang, Liang-Jie Hong, Yuan-Qiang Lu

**Affiliations:** ^1^Department of Emergency Medicine, The First Affiliated Hospital, School of Medicine, Zhejiang University, Hangzhou, 310003 Zhejiang, China; ^2^Department of Geriatric Medicine, The First Affiliated Hospital, School of Medicine, Zhejiang University, Hangzhou, 310003 Zhejiang, China; ^3^Zhejiang Provincial Key Laboratory for Diagnosis and Treatment of Aging and Physic-chemical Injury Diseases, The First Affiliated Hospital, School of Medicine, Zhejiang University, Hangzhou, 310003 Zhejiang, China; ^4^Zhejiang University, Hangzhou City, Zhejiang Province, China

## Abstract

**Objectives:**

The primary target is to reveal whether the resuscitation with hypertonic saline (HTS) or hydroxyethyl starch (HES) would have different effects on the myeloid-derived suppressor cell (MDSC) count and monocytic MDSC (M-MDSC)/granulocytic/neutrophilic MDSC (G-MDSC) rate in the peripheral blood, spleen, and bone marrow nucleated cells (BMNC) in a controlled hemorrhagic shock mouse model under secondary *Escherichia coli* bacterial infection attack, comparing to resuscitation with normal saline (NS) in 72 hours.

**Method:**

After hemorrhagic shock with bacteremia, which is induced by *Escherichia coli* bacterial infection attack, comparing to resuscitation with normal saline (NS) in 72 hours. *Method*. After hemorrhagic shock with bacteremia, which is induced by *Escherichia coli* 35218 injection, the mice were distributed into control, NS, HTS, and HES groups. The peripheral blood nucleated cells (PBNC), spleen single-cell suspension, and bone marrow nucleated cells were collected. The flow cytometry was used to detect the MDSC, M-MDSC, and G-MDSC.

**Result:**

In PBNC, after resuscitation with NS, the MDSC was continuously higher, while the rate of M-MDSC/G-MDSC were continuously lower (*P* < 0.05). In HTS, the MDSC varied, higher at 24 and 72 hours (*P* < 0.05). In HTS, the MDSC varied, higher at 24 and 72 hours (*P* < 0.05). In HTS, the MDSC varied, higher at 24 and 72 hours (*P* < 0.05). In HTS, the MDSC varied, higher at 24 and 72 hours (*P* < 0.05). In HTS, the MDSC varied, higher at 24 and 72 hours (*P* < 0.05), the M-MDSC/G-MDSC were continuously lower (*P* < 0.05). In the spleen, resuscitation with HTS, the M-MDSC/G-MDSC were continuously lower (*P* < 0.05). In BMNC, after resuscitation with HES, the M-MDSC/G-MDSC were lower at 24 and 72 hours (*P* < 0.05).

**Conclusion:**

In mouse hemorrhagic shock model with bacterial infection, the resuscitation with NS, HTS, or HES induced difference changes in MDSC and M-MDSC/G-MDSC, which were time-dependent and organ-specific. Resuscitation with crystalloid, like NS or HTS, showed longer effects on the MDSC and M-MDSC/G-MDSC in peripheral blood; while HTS has a longer effect on M-MDSC/G-MDSC in the spleen, HES has a stronger impact on the differentiation regulation of MDSC to G-MDSC in the bone marrow.

## 1. Introduction

Massive blood loss and remarkable microcirculation decrease are principal characteristics of hemorrhagic shock, which are usually inevitably followed by the systemic inflammation responses [1, 2]. The immune responses such as systemic inflammatory response syndrome (SIRS) could flow the body with inflammatory mediator, destroy the gut barrier function, and diminish the antimicrobial activity of phagocytic cells. The secondary bacterial infection is often the major threat for hemorrhagic shock. Myeloid-derived suppressor cells (MDSCs) belong to a heterogeneous group of myeloid cells that could suppress the function of T cells and natural killer (NK) cells. The immunosuppressive activities of MDSC in cancer, auto-immune disease, and some bacterial and viral infections have been widely studied [3, 4]. However, the detailed regulation and function of MDSC in hemorrhagic shock with bacterial infection have not been fully understood. In the hemorrhagic shock therapy, fluid resuscitation is a core stone. The crystalloid solutions like normal saline (NS), hypertonic saline (HTS), and the colloidal solution hydroxyethyl starch (HES) are the most commonly used in clinic and research [5, 6]. Besides the improvement in hemodynamic parameters, studies have also found the regulating effect of HTS and HES in systemic inflammation responses after hemorrhagic shock [7–11]. In previous research, our results already implied that compared to resuscitation with NS or HES in the rat model of hemorrhagic shock, HTS could notably elevate the MDSC in peripheral circulation in early stage after hemorrhagic shock [12]. Thus, in this article, we constructed a mouse model of controlled hemorrhagic shock with secondary *Escherichia coli* (*E. coli*) bacterial infection attack, then resuscitated these mice with single NS, HTS, and HES, respectively. We studied the distribution and subphenotypes of MDSC in the peripheral blood, spleen, and bone marrow nucleated cells (BMNC) in 72 hours after resuscitation. The primary goal aimed to reveal whether the resuscitation with HTS or HES has different effects on the MDSC count and M-MDSC/G-MDSC rate in the peripheral blood, spleen, and BMNC in a controlled hemorrhagic shock with secondary *Escherichia coli* bacterial infection attack.

## 2. Method

### 2.1. Animals

Male BALB/c mice, 15~20 weeks of age, weighing 24~30 g, were obtained from the Laboratory Animal Centre of Medical Institute of Zhejiang Province. The mice were fed with food and water *ad libitum* in specified-pathogens free (SPF) environment. The animal experiments, which followed the principles of the criteria outlined in the Guide for the Care and Use of Laboratory Animals, were approved by the Ethics Committee of the First Affiliated Hospital, Zhejiang University.

### 2.2. Experimental Protocol

After anesthetized with 0.04 mg/g pentobarbital (i.p.), the mouse got a first cut about 2 cm incision on the abdominal medialline, then was dissected layer by layer until the small intestine was exposed. Then, pushed the small intestine aside carefully to expose the inferior vena cava. Inferior vena cava puncture was made under direct sight by a 0.5 mm external diameter needle tip. An adult mouse has a circulating blood volume of about 1.5-2.5 ml (6-8% of the body weight, or 0.06-0.08 ml/g); age and obesity are the main influencing factors. The total blood volume (0.03 ml/g) was drawn slowly in 30 minutes to establish the controlled hemorrhagic shock model. It is estimated that the blood loss was close to 50% of total circulating blood volume. Following with 30-minute observation after the closing of abdominal incision, the resuscitation fluid NS, HTS, and HES were injected at a constant speed though the inferior vena cava by volumetric infusion pump in half hour, respectively. The wound was sutured if no blood leak was present. Then, the mice were observed under anesthesia for 2 hours; *E. coli* 35218 was injected to the caudal vein with the concentration of 1.0 × 10^5^ CFU/g after that. This bacterial injection dosage was given according to the experimental results and references [13, 14]. The mice were sacrificed in batches at three observation time points, 24, 48, and 72 hours after bacteria injection. The spleen, bone marrow, and peripheral blood were taken for cell harvest. All the procedures were carried out in a sterile environment.

### 2.3. Grouping of Animals

Eighty mice were randomly divided into control (*n* = 8), NS (*n* = 24), HTS (*n* = 24), and HES (*n* = 24) groups. The mice in control group only received anesthesia without any surgery operation. The operative procedures and dosage of bacteria injection were all the same, except the different resuscitation fluids among the other three groups. The NS group used 0.9% (*w*/*v*) NaCl about 3 times the blood loss volume. HTS group used 3% (*w*/*v*) NaCl solution with the dose of 1 : 1 volume to shed blood. HES group used hydroxyethyl starch (130/0.4) at the same volume of blood loss. The NS, HTS, and HES groups were divided into 24 hours (*n* = 8), 48 hours (*n* = 8), and 72 hours (*n* = 8) subgroup, respectively, according to the sacrificed time. All the operating process and fluid resuscitation principles are according to the directions, literature, and previous studies.

### 2.4. Collection of Sample

Heparin had been added to all the sample to prevent thrombogenesis before cell harvest. The monoplast suspension of the spleen, peripheral blood nucleated cells (PBNC), and bone marrow nucleated cells (BMNC) was prepared according to the protocol. RBC Lysis Buffer was used to remove the red blood cells from the samples.

### 2.5. Flow Cytometric Analysis

The monoclonal antibody and their isotype control were purchased from eBioscience, Inc. The main fluorescence-labelled antibodies are shown as follows: anti-mouse CD11b FITC antibodies (Catalog Number: 11-0112), anti-mouse Ly-6C PE antibodies (Catalog Number: 12-5932), and anti-mouse Ly-6G (Gr-1) PerCP-Cyanine5.5 antibodies (Catalog Number: 45-5931). The hydroxyethyl starch (130/0.4) was bought from Fresenius Kabi Deutschland GmbH. The FACSCalibur (Beckman-Coulter, USA) was used to flow cytometric analysis. The cells marked with CD11b^+^Ly6G^+^Ly6C^low/int^ were granulocytic/neutrophilic MDSC (G-MDSC); the monocytic MDSC (M-MDSC) were the cells with CD11b^+^Ly6G^−^Ly6C^high^. The MDSC are CD11b^+^ cells labeled with either Ly6G^+^ or Ly6C^high^.

### 2.6. Statistical Analysis

Independent sample *t*-test between control and NS, control and HTS, and control and HES in each time subgroups was conducted to clarify the statistical significance. A *P* value of <0.05 was considered statistically significant.

## 3. Results

### 3.1. Characteristics of Mice Model

All the male BALB/c mice in our study survived successfully until the sacrifice time points. There were no significant differences among the four groups with regard to age or weight (both *P* > 0.05).

After resuscitation with three different fluids under the coefficient of bacterial and hemorrhagic shock, the distribution of MDSC and the ratio of M-MDSC and G-MDSC in mice were investigated at three time points.

### 3.2. Distribution and Subphenotypes of MDSC in PBNC after Fluid Resuscitation

**Figure 1:**
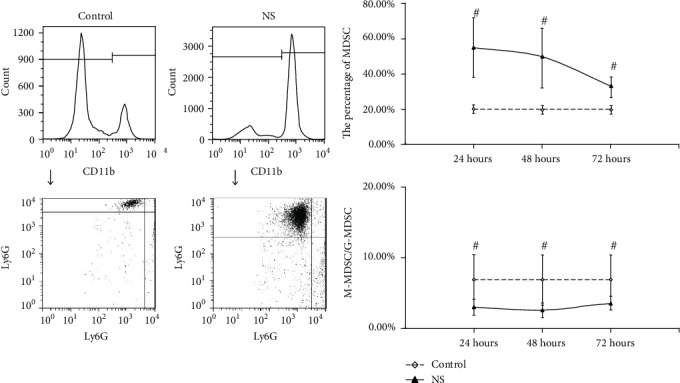
The count of MDSC and ratio of M-MDSC/G-MDSC in PBNC after fluid resuscitation by NS. #: compared to control, *P* < 0.05; MDSC: myeloid-derived suppressor cell; M-MDSC: monocytic MDSC; G-MDSC: granulocytic/neutrophilic MDSC; PBNC: peripheral blood nucleated cells; NS: normal saline.

**Figure 2:**
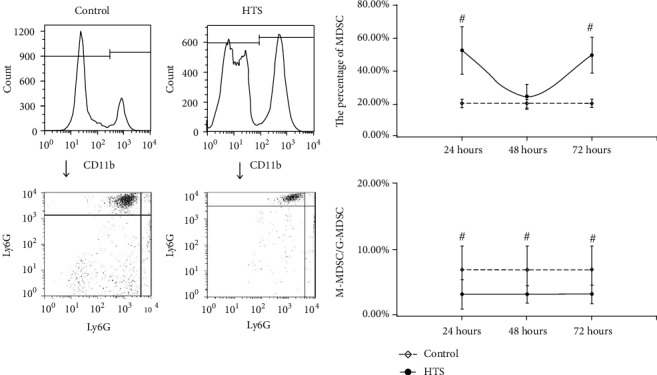
The count of MDSC and ratio of M-MDSC/G-MDSC in PBNC after fluid resuscitation by HTS. #: compared to control, *P* < 0.05; MDSC: myeloid-derived suppressor cell; M-MDSC: monocytic MDSC; G-MDSC: granulocytic/neutrophilic MDSC; PBNC: peripheral blood nucleated cells; HTS: hypertonic saline.

**Figure 3:**
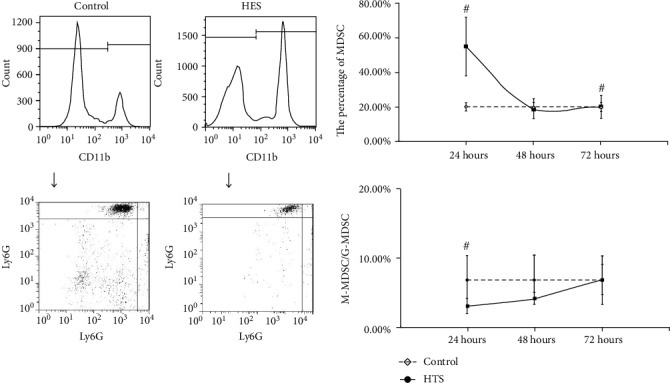
The count of MDSC and ratio of M-MDSC/G-MDSC in PBNC after fluid resuscitation by HES. #: compared to control, *P* < 0.05; M-MDSC: monocytic MDSC; G-MDSC: granulocytic/neutrophilic MDSC; PBNC: peripheral blood nucleated cells; HES: hydroxyethyl starch.

For the MDSC in PBNC, after resuscitation with NS, the MDSC counts were continuously higher than control, while the rate of M-MDSC/G-MDSC were continuously lower than control, at 24 hours, 48 hours, and 72 hours with statistical significance (*P* < 0.05) (Figure 1). In group resuscitation with HTS, the MDSC counts varied depending on the length of observation time, that is, higher than control at 24 hours and 72 hours with statistical significance (*P* < 0.05), but shown no statistical significance (*P* > 0.05) at 48 hours. However, the rate of M-MDSC/G-MDSC were continuously lower than control in total times with statistical significance (*P* < 0.05) (Figure 2). The significant change (*P* < 0.05) of the MDSC count and M-MDSC/G-MDSC after resuscitation with HES appears at 24 hours, which was higher than control in MDSC count and lower than control in the M-MDSC/G-MDSC rate, and there were no statistical significance in 48 hours and 72 hours (Figure 3).

### 3.3. Distribution and Subphenotypes of MDSC in the Spleen after Fluid Resuscitation

**Figure 4:**
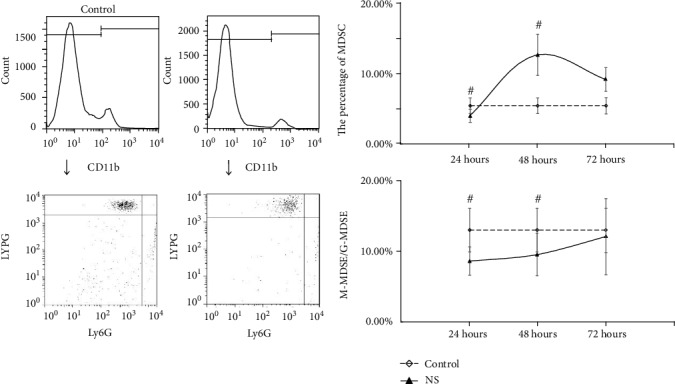
The count of MDSC and ratio of M-MDSC/G-MDSC in the spleen after fluid resuscitation by NS. #: compared to control, *P* < 0.05; MDSC: myeloid-derived suppressor cell; M-MDSC: monocytic MDSC; G-MDSC: granulocytic/neutrophilic MDSC; NS: normal saline.

**Figure 5:**
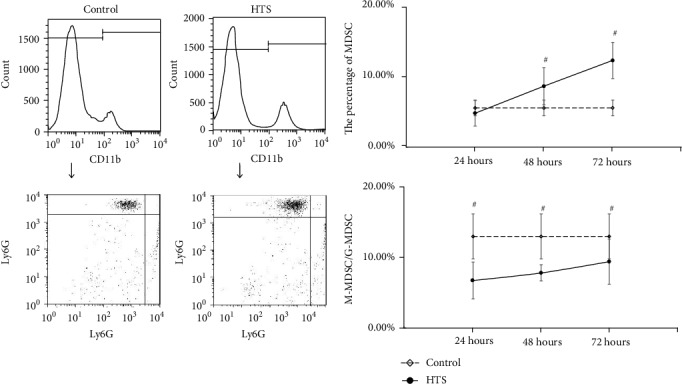
The count of MDSC and ratio of M-MDSC/G-MDSC in the spleen after fluid resuscitation by HTS. #: compared to control, *P* < 0.05; MDSC: myeloid-derived suppressor cell; M-MDSC: monocytic MDSC; G-MDSC: granulocytic/neutrophilic MDSC; HTS: hypertonic saline.

**Figure 6:**
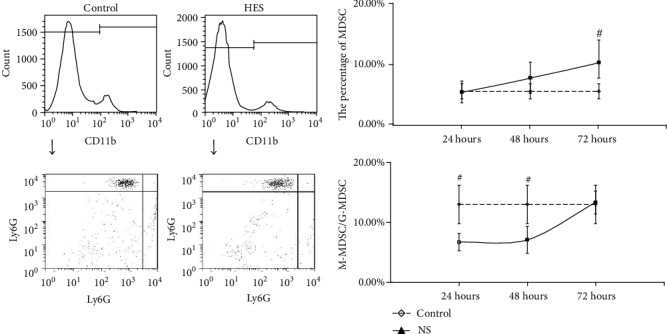
The count of MDSC and ratio of M-MDSC/G-MDSC in the spleen after fluid resuscitation by HES. #: compared to control, *P* < 0.05; MDSC: myeloid-derived suppressor cell; M-MDSC: monocytic MDSC; G-MDSC: granulocytic/neutrophilic MDSC; HES: hydroxyethyl starch.

For the MDSC in the spleen, resuscitation with NS induced lower MDSC count in the spleen at 24 hours (*P* < 0.05), but increased significantly to higher than control at 48 hours and 72 hours (*P* < 0.05). The M-MDSC/G-MDSC were lower than control at 24 hours and 48 hours with significance (*P* < 0.05), but shown no statistical difference at 72 hours (*P* > 0.05) (Figure 4). For resuscitation with HTS, the MDSC count were similar with control at 24 hours (*P* > 0.05), but higher than control at 48 hours and 72 hours (*P* < 0.05), while M-MDSC/G-MDSC were continuously lower than control in three times with statistical significance (*P* < 0.05) (Figure 5). For resuscitation with HES, the obviously different of MDSC count only appeared at 72 hours (*P* < 0.05), while the rate of M-MDSC/G-MDSC were lower than control at 24 hours and 48 hours (*P* < 0.05), with no statistical significance at 72 hours (*P* > 0.05) (Figure 6).

### 3.4. Distribution and Subphenotypes of MDSC in BMNC after Fluid Resuscitation

**Figure 7:**
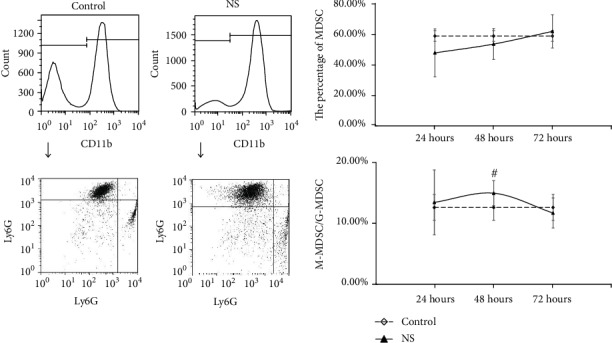
The count of MDSC and ratio of M-MDSC/G-MDSC in BMNC after fluid resuscitation by NS. #: compared to control, *P* < 0.05; MDSC: myeloid-derived suppressor cell; M-MDSC: monocytic MDSC; G-MDSC: granulocytic/neutrophilic MDSC; BMNC: suspension and bone marrow nucleated cells; NS: normal saline.

**Figure 8:**
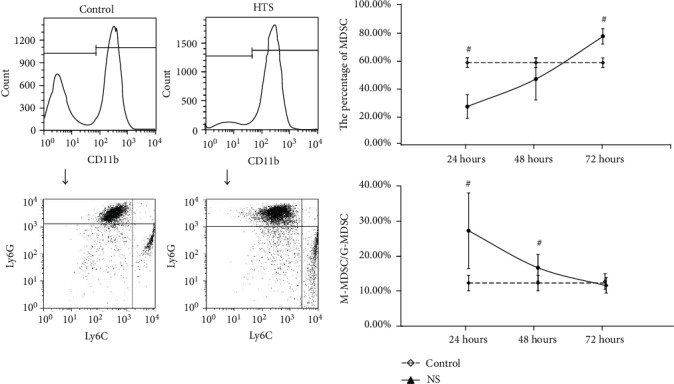
The count of MDSC and ratio of M-MDSC/G-MDSC in BMNC after fluid resuscitation by HTS. #: compared to control, *P* < 0.05; MDSC: myeloid-derived suppressor cell; M-MDSC: monocytic MDSC; G-MDSC: granulocytic/neutrophilic MDSC; BMNC: suspension and bone marrow nucleated cells; HTS: hypertonic saline.

**Figure 9:**
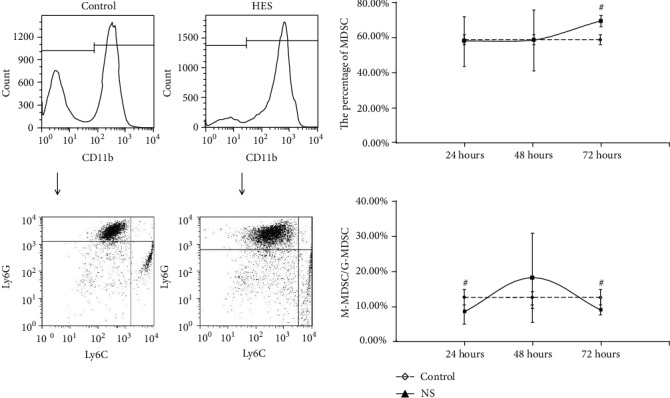
The count of MDSC and ratio of M-MDSC/G-MDSC in BMNC after fluid resuscitation by HES. #: compared to control, *P* < 0.05; MDSC: myeloid-derived suppressor cell; M-MDSC: monocytic MDSC; G-MDSC: granulocytic/neutrophilic MDSC; BMNC: suspension and bone marrow nucleated cells; HES: hydroxyethyl starch.

For the MDSC in BMNC, after resuscitation with NS, the variation of MDSC count and M-MDSC/G-MDSC shown no statistical significance at three times (*P* > 0.05); only the M-MDSC/G-MDSC rate at 48 hours were higher than control (*P* < 0.05) (Figure 7). After resuscitation with HTS, the MDSC count was lower than control at 24 hours but higher than control at 72 hours with statistical significance (*P* < 0.05). The M-MDSC/G-MDSC were all higher than control at 24 hours and 48 hours with statistical significance (*P* < 0.05) (Figure 8.). In group resuscitation with HES, no differences had been found in MDSC count at 24 hours and 48 hours (*P* > 0.05), but the MDSC count was higher than control at 72 hours (*P* < 0.05). The M-MDSC/G-MDSC were all lower than control at 24 hours and 72 hours with statistical significance (*P* < 0.05), while the differences at 48 hours shown no statistical significance (*P* > 0.05) (Figure 9).

## 4. Discussion

Hemorrhagic shock often followed by severe immune disorders, which could increase the risk of bacterial infection [15]. MDSC can influence the innate and adaptive immune systems to defend against the invading microbes; thus, the elevated MDSC plays crucial role in the chronicity and persistency of infection [3, 4, 16–21]. In previous rat hemorrhagic shock model, we have found that resuscitation with HTS induced the dramatical migration and redistribution of MDSC from the bone marrow to peripheral circulation, comparing with the resuscitation with NS or HES at the early stage of shock [12].

In this research, we prolonged the observation time to 72 hours in hemorrhagic shock mouse model with bacterial infection, hoping to evaluate the influence of different means of fluid resuscitation on the distribution and subphenotypes of MDSC. We displayed that resuscitation with NS, HTS, and HES has different effects on MDSC distribution and subphenotypes. It was worth noting that the MDSC count and M-MDSC/G-MDSC in peripheral blood of NS and HTS group were still higher than control after 72 hours, but at the same time, no such results were supported by statistics in HES groups. This implied that crystalloid might had a longer effect on the MDSC in peripheral blood. In addition, HTS has a longer effect on the distribution regulation of MDSC in the spleen, and HES has a delayed but stronger impact on the differentiation regulation of MDSC in the bone marrow, because of the differential inclination to G-MDSC in the spleen of HTS and in BMNC of HES groups was found at 72 hours. M-MDSC and G-MDSC, two major MDSC subsets, differ in their immunosuppressive mechanisms, cell phenotypes, gene expression profiles, distributions, and the responses to different bacteria [22–24]. G-MDSC is often been found as the predominant subset in chronic Gram-positive bacterial infection, while M-MDSC is usually in the chronic bacterial infection and has the capability to differentiate into G-MDSC [21, 23, 25, 26]. They would lose their immunosuppressive function, and differentiate into mature cells, according to different stimulus [27, 28].

MDSC plays a paradoxical role in sepsis and trauma [29]. Researches based on cancers have proved its immunosuppressive function in many kinds of neoplasms. Other studies also verified their inhibit function in chronic infection and autoimmune diseases. However, recent studies suggest the proliferation of MDSC in some acute inflammatory processes, such as burns and sepsis, could enhance immune surveillance and innate immune responses. These indicated the positive effect of MDSC in infection control. Study also implied MDSC has divergent gene expression profiles and functions in early and late sepsis. MDSC could express nitric oxide synthase and proinflammatory cytokines which both have proinflammatory function at early stage; conversely, at late stage, it expresses arginase, interleukin 10 (IL-10), and transforming growth factor *β* (TGF-*β*) which are responsible for immunosuppressive function. Therefore, the main emphasis of our research is to understand the distribution and differentiation of MDSC in hemorrhagic shock associated with bacteremia after resuscitation with NS, HTS, or HES.

HTS has been proved to transiently increase the level and osmolality of serum sodium, which associates with immunomodulatory effects [7, 30, 31]. Although the elevation of serum sodium begins to recede half or one hour later after HTS resuscitation, its effect on immune function still lasts for up to 24 hours. The pharmacokinetics also shows that HES would be no residual 24 hours after resuscitation. Except the hemodynamic properties, HTS and HES both have other effects [5, 11, 32]. HTS can reduce endothelial and tissue edema, improve microcirculation, inhibit the expression of adhesion molecule in endothelial cells, suppress the activation of neutrophil, alter inflammatory cytokine production, and attenuate oxidative stress [33–35]. HES also exhibits the anti-inflammatory and amelioration of oxidative stress functions [36, 37]. Our study demonstrated the time-dependent distributions and subphenotypes of MDSC in hemorrhagic shock mouse model associated with bacteremia after resuscitation with NS, HTS, or HES. Some influences of resuscitation fluids may last for at least 72 hours.

The proliferation, activation, and immunosuppressive mechanisms of MDSC base on cell phenotype, pathological type, and the species studied. The primary signaling pathway responsible for its proliferation and differentiation is STAT3, which can be activated by granulocyte-macrophage colony stimulating factor (GM-CSF), VEGF, or IL-6. The factors and signaling pathways involved in MDSC function activation include STAT3, NF-*κ*B, TNF-*α*, IL-1*β*, MyD88, and toll-like receptor signaling [38]. HTS can increase the release of endogenous IL-10 by activating STAT3 in the process of ischemia-reperfusion injury, which is the major pathologic change in hemorrhagic shock [39]. The other studies based on different animal models present that HTS and HES both could block the increase of TNF-*α*, IL-1*β*, IL-6, or GM-CSF. Research on burn mouse model shows that resuscitation with HTS could augment NF-*κ*B, TLR4, MyD88, and pp38 expressions, decrease the apoptosis of inflammatory cells, and enhance bacterial clearance and bacterial killing activities [5, 9, 30, 33, 35, 36, 40–44]. Resuscitation with HES also exhibits an anti-inflammatory effect in the intestinal injury after hemorrhagic shock, by dropping TNF-*α*, IL-6 expressions, and NF-*κ*B activation in ileum. In patients undergoing coronary surgery, resuscitation with HES has a higher level of TNF-*α* compared to a balanced electrolyte crystalloid solution 2 hours after surgery [8, 36, 37, 45]. All the evidences above reveal that the resuscitation fluids could either directly or indirectly alter the expression of cytokines and activation of signaling pathways, and these changes would move outward to affect the MDSC and other immune system.

In conclusion, the detailed mechanism and function of MDSC in hemorrhagic shock with or without bacteremia were still unclear. Prolonging the observation time, adding cellular phenotype study, adding specific molecular marker, designing function experiment in vitro or vivo cell, and adding cell track in vivo and signaling pathway could help us better understand the above questions.

The main discoveries in this article are that when associated with *E. coli* bacteremia, hemorrhagic shock resuscitation with NS, HTS, and HES could induce the difference changes in MDSC count and M-MDSC/G-MDSC rate, which were time-dependent and organ-specific. All three fluids could increase the distribution of MDSC in peripheral blood and enhance the differentiation trend of MDSC to G-MDSC in the spleen at the end of 24 hours. All of the three fluids could expand the counts of MDSC in the spleen at 72 hours. Resuscitation with crystalloid, either NS or HTS, showed a longer effect on the MDSC count and M-MDSC/G-MDSC in peripheral blood, which HTS and HES may have longer impacts on the MDSC count in the bone marrow. HTS have a longer effect on the distribution regulation of MDSC in the spleen, while HES may have a stronger impact on the differentiation regulation of MDSC to G-MDSC in the bone marrow. Further researches need to uncover the mechanism underlying these phenomena in such traumatic stress.

## Data Availability

The [DATA TYPE] data used to support the findings of this study are included within the article.
